# Dopamine-Mediated Circadian and Light/Dark-Adaptive Modulation of Chemical and Electrical Synapses in the Outer Retina

**DOI:** 10.3389/fncel.2021.647541

**Published:** 2021-05-05

**Authors:** Manvi Goel, Stuart C. Mangel

**Affiliations:** Department of Neuroscience, Ohio State University College of Medicine, Columbus, OH, United States

**Keywords:** GABAA receptors, gap junctions, receptive field surround, circadian rhythms, cone and rod photoreceptor cells, bipolar cells, horizontal cells, dopamine D1 and D4 receptors

## Abstract

The vertebrate retina, like most other brain regions, undergoes relatively slow alterations in neural signaling in response to gradual changes in physiological conditions (e.g., activity changes to rest), or in response to gradual changes in environmental conditions (e.g., day changes into night). As occurs elsewhere in the brain, the modulatory processes that mediate slow adaptation in the retina are driven by extrinsic signals (e.g., changes in ambient light level) and/or by intrinsic signals such as those of the circadian (24-h) clock in the retina. This review article describes and discusses the extrinsic and intrinsic modulatory processes that enable neural circuits in the retina to optimize their visual performance throughout day and night as the ambient light level changes by ~10 billion-fold. In the first synaptic layer of the retina, cone photoreceptor cells form gap junctions with rods and signal cone-bipolar and horizontal cells (HCs). Distinct extrinsic and intrinsic modulatory processes in this synaptic layer are mediated by long-range feedback of the neuromodulator dopamine. Dopamine is released by dopaminergic cells, interneurons whose cell bodies are located in the second synaptic layer of the retina. Distinct actions of dopamine modulate chemical and electrical synapses in day and night. The retinal circadian clock increases dopamine release in the day compared to night, activating high-affinity dopamine D_4_ receptors on cones. This clock effect controls electrical synapses between rods and cones so that rod-cone electrical coupling is minimal in the day and robust at night. The increase in rod-cone coupling at night improves the signal-to-noise ratio and the reliability of very dim multi-photon light responses, thereby enhancing detection of large dim objects on moonless nights.Conversely, maintained (30 min) bright illumination in the day compared to maintained darkness releases sufficient dopamine to activate low-affinity dopamine D_1_ receptors on cone-bipolar cell dendrites. This non-circadian light/dark adaptive process regulates the function of GABA_A_ receptors on ON-cone-bipolar cell dendrites so that the receptive field (RF) surround of the cells is strong following maintained bright illumination but minimal following maintained darkness. The increase in surround strength in the day following maintained bright illumination enhances the detection of edges and fine spatial details.

## Introduction

A major goal of research on the retina and other brain regions is to understand the mechanisms that underlie the modulation of neuronal properties (e.g., transmitter receptors, ion channels, gap junction channels, transporters, second messenger pathways), synaptic communication, and neural network activity in response to changes in the environment or physiological conditions. Most regions of the central nervous system including the retina undergo relatively slow alterations in neural signaling in response to gradual changes in physiological conditions, as when activity changes to rest, or in response to gradual changes in environmental conditions, as when day changes into night. The retinal response to daily changes in the visual environment due to rotation of the Earth are particularly intriguing because the ambient (background) light level changes a great deal (~10 billion-fold) and because visual performance needs at midday are very different than those on a moonless night. Survival requires high acuity at midday and sensitivity to very dim objects on moonless nights. As occurs elsewhere in the brain, the modulatory processes that mediate slow adaptation in the retina are driven by extrinsic signals (e.g., changes in ambient light level) and/or by intrinsic signals such as those of the circadian (24-h) clock in the retina.

**Figure 1:**
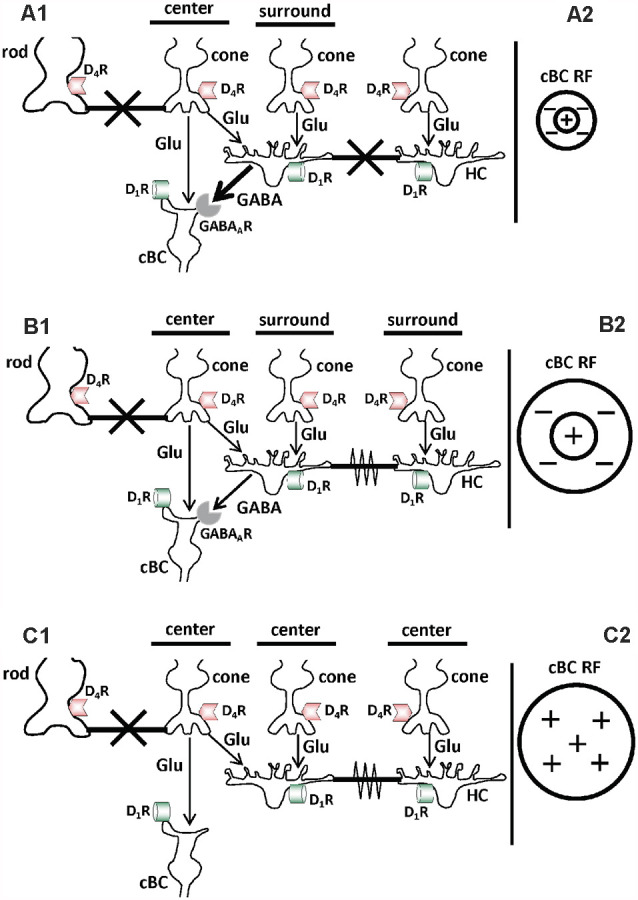
Schematic diagrams showing that in the day the background (ambient) light level modulates chemical and electrical synaptic signaling in the first synaptic layer of the retina (outer plexiform layer) by regulating activation of D_1_ receptors on the dendrites of cone bipolar cells and horizontal cells (HCs). **(A1,B1,C1)** Cones use glutamate (Glu) to signal the dendrites of cone bipolar cells (cBCs) and horizontal cells (HCs). The dendrites of cBCs and HCs have both dopamine D_1_ receptors (D_1_Rs) and GABA_A_Rs, and rod and cone synaptic terminals have dopamine D_4_Rs. Gap junctions, which can function as electrical synapses when open, are located between rod and cone synaptic terminals and between HCs. Evidence has shown that in the day gradual changes in the background (ambient) light level alter GABA_A_R-mediated chemical synaptic transmission from HCs to cBCs, and electrical synaptic signaling between HCs. **(A1,A2)** Following maintained (>30 min) bright illumination, dopamine release is high and endogenous activation of D_1_Rs on cBCs and HCs is strong, increasing cAMP/PKA in the cells. This in turn uncouples the gap junctions between HCs and increases the expression of functional GABA_A_Rs on the dendrites of cBCs **(A1)**. In addition, gap junctions between rods and cones are closed due to endogenous activation of their D_4_Rs **(A1)**. As a result, the GABA_A_R-mediated receptive field (RF) surround of cBCs is strong. In addition, cBC surrounds are small, because HCs, which are uncoupled, signal the size of cBC surrounds **(A2)**. In addition, the HC surround signal, which is opposite in polarity to the cBC center and small in spatial extent, provides lateral inhibition that effectively reduces the size of the cBC center **(A2)**. **(B1,B2)** Following maintained dim illumination in the day, dopamine release is lower and endogenous activation of D_1_Rs on cBCs and HCs is weaker, decreasing cAMP/PKA in the cells. This is turn recouples the gap junctions between HCs and decreases the expression of functional GABA_A_Rs on the dendrites of cBCs **(B1)**. In addition, gap junctions between rods and cones are closed due to endogenous activation of their D_4_Rs **(B1)**. As a result, the receptive field surround of cBCs is larger than following maintained bright illumination because HCs are coupled **(B2)**. In addition, the HC surround signal, which is larger in spatial extent than following bright illumination, provides lateral inhibition that is less effective in reducing the size of the cBC center **(B2)**. **(A2,B2)** Although only one cone is depicted providing direct input to the cBC (so as not to overclutter the figure), a more accurate portrayal would show two cones providing input. Following bright illumination **(A2)**, only one cone provides effective input to the cBC due to the strength and small size of the HC inhibitory surround signal. As a result, the cBC center size is small. In contrast, following dim illumination **(B2)**, both cones provide effective input to the cBC due to the reduced strength and larger size of the HC inhibitory surround signal. As a result, the cBC center size is larger. **(C1,C2)** Following maintained darkness in the day, dopamine release is much lower and endogenous activation of D_1_Rs on cBCs and HCs is minimal, greatly decreasing cAMP/PKA in the cells. As a result, gap junctions between HCs are open and the dendrites of cBCs do not express GABA_A_Rs **(C1)**. In addition, gap junctions between rods and cones are closed because the retinal clock releases enough dopamine to activate their D_4_Rs, which are more sensitive to dopamine than D_1_Rs (see below; **C1**). As a result, the RF center of cBCs is large but the surround is minimal or absent (**C2**). **(A1–C1)** thick black horizontal bars with large “X”: closed gap junctions; thick black horizontal bars with zigzag line: open gap junctions. **(A2–C2)** Plus signs (+): RF center of ON-cBCs; Minus signs (−): RF surround of ON-cBCs.

This review will focus on extrinsic and intrinsic signal modulation in the first synaptic layer (or outer plexiform layer) of the retina. In this portion of the retina, cone photoreceptor cells initiate visual processing when the ambient (background) illumination is bright and rod photoreceptor cells initiate visual processing when it is very dim or dark (Mangel and Ribelayga, 2010; Dowling, 2012). Cones form gap junctions with rods and use glutamate to signal the dendrites of cone bipolar cells (cBCs) and horizontal cells (HCs; Figure 1). HCs also form gap junctions with each other. Distinct extrinsic and intrinsic modulatory processes in the outer plexiform layer are mediated by long-range dopamine feedback from the second synaptic layer (inner plexiform layer). Both light and the retinal circadian clock increase dopamine release from dopaminergic cells (Witkovsky, 2004; Dowling, 2012), interneurons whose cell bodies are located in the inner nuclear layer. Distinct actions of dopamine via activation of specific dopamine receptor types on cones, rods, cBCs and HCs (Figure 1) modulate chemical and electrical synapses in day and night. This review describes and discusses the dopamine-mediated extrinsic and intrinsic modulatory processes that enable neural circuits in the outer retina to optimize their visual performance throughout day and night as the ambient illumination gradually changes.

## Dopamine Function in The Outer Retina

### Dopamine Receptors, Dopamine Release, and Dopamine Pathways in the Retina

**Figure 2:**
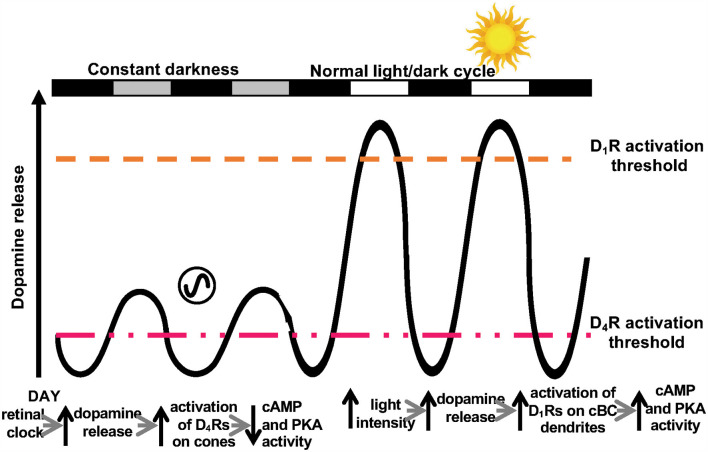
Two dopamine receptor systems in the retina. Schematic representation of the dual control of dopamine release by the retinal circadian clock and light in the fish retina, which activate D_4_Rs and D_1_Rs, respectively. Although the retinal clock releases less dopamine in the day than bright illumination, circadian clock-induced dopamine release in the day is sufficient to activate cone D_4_Rs (but not D_1_Rs on cBCs and HCs), because D_4_Rs are ~500 times more sensitive to dopamine than D_1_Rs. As a result, cAMP/PKA in cones is low in the day. In constant darkness at night, dopamine levels are lower than in the day and not sufficient to activate cone D_4_Rs. As a result, cAMP/PKA in cones increases at night. The circadian rhythm in dopamine release is due to the inhibitory action of melatonin on dopamine release. The retinal clock increases melatonin synthesis and release to a greater extent at night than in the day, which results in a circadian rhythm in dopamine release that is opposite in phase (i.e., higher in the day than at night). During the subjective day (SD), melatonin levels are low and as a result, so is its inhibitory action on dopamine release. Consequently, during the subjective day, extracellular dopamine levels increase sufficiently to activate D_4_ receptors, but insufficiently to activate D_1_ receptors. During the regular light/dark cycle, daylight increases dopamine release sufficiently to activate D_1_ receptors. Modified from Ribelayga and Mangel (2003).

Dopamine binds and acts through two dopamine receptor families, i.e., the D1 receptor family that includes D_1_ and D_5_ receptors and the D2 receptor family that includes D_2_, D_3_, and D_4_ receptors. All dopamine receptors are metabotropic, G protein coupled receptors, whose activation either enhances or blocks downstream signaling pathways. Activation of the D1 receptor family (G_s_ receptor type) increases adenylyl cyclase activity, whereas activation of the D2 receptor family (G_i_ receptor) decreases adenylyl cyclase activity (Figure 2). In addition, the D2 receptor family is 100–500 times more sensitive to dopamine than the D_1_ receptor family (Kebabian and Calne, 1979; Missale et al., 1998). Dopamine D_4_ receptors are the most sensitive dopamine receptor type, binding endogenous dopamine in the low nM range (Figure 2). The difference in sensitivity of the various dopamine receptor subtypes may be an under-appreciated, but nonetheless, fundamental functional characteristic of these receptors.

In the outer plexiform layer, rods and cones express D_4_Rs (Figure 1), but not dopamine D_1_Rs (Nguyen-Legros et al., 1999; Witkovsky, 2004; Iuvone et al., 2005). Conversely, the dendrites of HCs and cBCs (both ON- and OFF-cBCs) express D_1_Rs but not D_4_Rs.

All vertebrate species have a single type of dopaminergic cell, which may contain several subtypes (Zhang et al., 2007). These cells, which in most species are called dopaminergic amacrine cells, and in other species dopaminergic interplexiform cells (Dowling and Ehinger, 1978) have cell bodies among amacrine cells in the inner nuclear layer. Dopaminergic interplexiform cells, which are found in fish and primates including humans, are so-called because one of their output processes travels to the outer plexiform layer where it makes synaptic contact with HCs and/or photoreceptors (Dowling and Ehinger, 1975). In contrast, these output processes in dopaminergic amacrine cells are shorter; in some species they barely leave the inner plexiform layer (e.g., rabbit) and in other species they travel a relatively short distance towards the outer retina (e.g., mouse). Both dopaminergic amacrine and interplexiform cells release dopamine onto other neurons from processes in the inner plexiform layer.

Interestingly, evidence suggests that dopamine reaches most retinal neurons *via* volume diffusion and not *via* direct synaptic contact (Ribelayga et al., 2002; Witkovsky, 2004). Moreover, dopamine and tyrosine hydroxylase, the rate limiting enzyme in dopamine synthesis, are observed throughout most, and possibly all, processes of dopaminergic cells, suggesting that dopamine may be synthesized and released all along the processes, and that dopamine release involves Na^+^-spiking (Dowling and Ehinger, 1978; Puopolo et al., 2001; Witkovsky, 2004). There is evidence that the synaptic terminals/release sites of dopaminergic cells express D_2_Rs (a member of the D2R family), which function as dopamine autoreceptors, i.e., activation of D_2_Rs decreases dopamine release (Harsanyi and Mangel, 1992; Wang et al., 1997). In addition, retinal ganglion cells express both D_1_ and D_2_ receptors (Veruki, 1997; Koulen, 1999; Ogata et al., 2012; Van Hook et al., 2012).

Dopaminergic amacrine and dopaminergic interplexiform cells receive inputs from rod and cone pathways as well as input from intrinsically photosensitive retinal ganglion cells (ipRGCs; Marshak, 2001; Dumitrescu et al., 2009; Qiao et al., 2016; Zhao et al., 2017). Dopaminergic amacrine cells exhibit a variety of response properties that make them functionally diverse. Dopaminergic cells receive both On and Off inhibitory responses from BCs *via* GABAergic and glycinergic amacrine cells in the inner plexiform layer (Qiao et al., 2016). In the presence of light, dopaminergic amacrine cells exhibit two kinds of light responses—On sustained and On transient responses (Zhang et al., 2007). In dim light, dopaminergic amacrine cells also receive inhibitory input from the rod pathway *via* glycinergic amacrine cells (Newkirk et al., 2013). Some On-BCs make ectopic synapses ontodopaminergic amacrine cells and ipRGCs in the Off sub-lamina of inner plexiform layer (Dumitrescu et al., 2009; Hoshi et al., 2009). Somatostatin amacrine cells also form inhibitory synapses on dopaminergic amacrine cells as well as ipRGCs through somatostatin sst2 and sst4 receptors, respectively (Vuong et al., 2015). ipRGCs also provide retrograde signals to dopaminergic amacrine cells that can eventually alter the visual responses retinal ganglion cells (Prigge et al., 2016). Sustained light responses in some dopaminergic neurons may be mediated by inputs from ipRGCs in the inner plexiform layer (Zhang et al., 2008). Conversely, dopamine also alters the visual responses of ipRGCs through D_1_Rs (Van Hook et al., 2012).

### Two Dopamine Receptor Systems in the Retina and Their Distinct Roles in Extrinsic and Intrinsic Modulatory Processes

Evidence suggests that there are two dopamine systems in the retina (Figure 2) that function in a complementary fashion. This idea arose from consideration of the extant literature on the circadian and non-circadian effects of dopamine in the retina and from measurements of the modulation of gap junction coupling between rods and cones, on the one hand, and between HCs on the other hand (Ribelayga and Mangel, 2003, 2007, 2010; Ribelayga et al., 2008). Taken together, these studies indicated that the retinal circadian clock, which increases dopamine release in the day by ~3× compared to night (Ribelayga et al., 2002; Witkovsky, 2004), acts through high-affinity D_4_Rs on rods and cones (Ribelayga et al., 2002, 2008, 2004; Witkovsky, 2004; Mangel and Ribelayga, 2010; Ribelayga and Mangel, 2010). In contrast, the increase in clock-mediated dopamine release in the day is not sufficient to affect coupling between cone HCs or between rod HCs (Ribelayga and Mangel, 2003, 2007, 2010). cHC-cHC coupling and rod HC-rod HC coupling are high in the dark in both day and night (i.e., coupling is not controlled by a circadian clock) and greatly reduced by bright illumination, which activates low-affinity D_1_Rs on the cells. Another dopamine-mediated example of neuromodulation in the retina that is not controlled by the retinal clock, but is regulated by the level of background illumination is the receptive field (RF) surround. Specifically, it has been shown recently that dopamine D_1_Rs mediate light-dark modulation of the strength of ON-cBC receptive field surrounds by regulating GABA_A_Rs on the dendrites of the cells (see below; Chaffiol et al., 2017).

These above observations of how dopamine acts as a neuromodulator in the retina thus provide evidence for the notion that there are two dopamine receptor systems in the retina. In one of these systems, dopamine, by activating high-affinity D_4_Rs, functions as an effector of an intrinsic molecular process (i.e., the retinal circadian clock). In the second system, the extracellular concentration of dopamine, by activating low-affinity D_1_Rs, constitutes a response to extrinsic changes in the visual environment (i.e., changes in the background illumination). We focus for the remainder of this review, on these dopamine-mediated extrinsic and intrinsic neuromodulatory processes, which involve these two dopamine receptor systems.

## Dopamine-Mediated Extrinsic Modulatory Processes in The Outer Retina

### Dopamine-Mediated Light-Dark Modulation of GABA_A_ Receptors and the Receptive Field Surround of ON-cBCs

A fundamental feature of most neurons in the visual, auditory, and somatosensory systems is that nearby sensory cells inhibit each other. This phenomenon, which is called lateral inhibition, was first reported by Hartline and his colleagues from observations of how adjacent photoreceptors in the horseshoe crab, *Limulus*, inhibited each other’s light responses (Thoreson and Mangel, 2012). A similar lateral inhibitory process in vertebrate retinas was reported decades ago in *in vivo* cat ganglion cells, which were found to exhibit a center-surround receptive field organization (Kuffler, 1953). Lateral or surround inhibition was found to improve spatial discrimination and the detection of edges. Interestingly, it was also reported many decades ago that the strength of receptive field surrounds of *in vivo* cat ganglion cells depends on the maintained light level, i.e., surrounds were the strongest following maintained bright illumination, became gradually weaker following a change to dark-adapted conditions, and eventually became minimal after ~30 min in the dark (Barlow et al., 1957; Barlow and Levick, 1969). In addition, as surrounds became weaker during the change to darkness, the receptive field center became larger in size (Troy and Shou, 2002), in agreement with the finding that surrounds laterally inhibit center light responses. It was also observed that the receptive field surround has three different states that depend on the background light level (see below). Similar findings have also been obtained in other species (e.g., rabbit ganglion cells; Muller and Dacheux, 1997).

**Figure 3:**
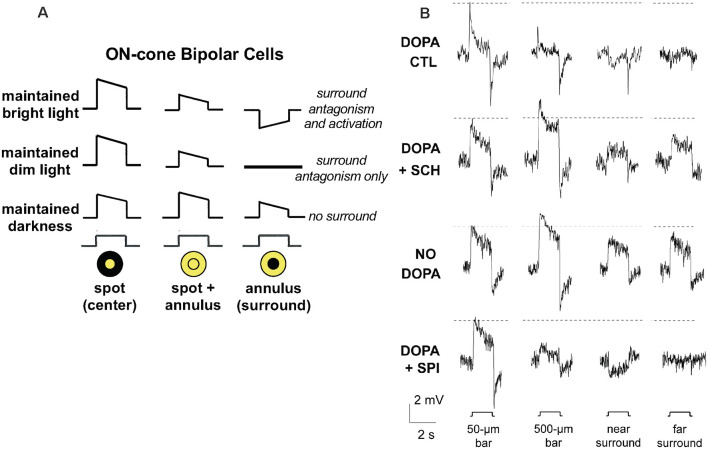
Effect of maintained background light level on receptive field surround of cone bipolar cells (cBCs) is mediated by dopamine D_1_Rs on cBC dendrites. **(A)** ON-cBC surround antagonism and surround activation are affected by changes in the maintained (30 min) background light level. At all background light levels ON-cBCs produce depolarizing responses to small centered spot stimuli (Column 1). ON-cBCs also produce surround responses that increase in strength and decrease in size as the maintained light level increases. Following maintained bright illumination, surround stimulation in the presence of center stimulation (spot and annulus or ring of light) reduces the center response size of ON-cBCs, a phenomenon known as “surround antagonism.” Also, ON-cBCs produce hyperpolarizing responses to surround stimulation alone (annulus of light) that are opposite in polarity to those produced by center (spot) stimulation alone, a phenomenon known as “surround activation.” Following maintained dim illumination, ON-cBCs exhibit surround antagonism but not activation. Following maintained darkness, ON-cBCs exhibit center responses but minimal surround. HCs (not shown) produce hyperpolarizing responses to all light stimuli at all light levels (i.e., HCs lack surrounds). At all light levels ON-ganglion cell center and surround light responses (not shown) are similar to those of ON-cBCs, but OFF-cBC center and surround responses (not shown) are opposite in polarity to those of ON-cBCs. **(B)** In whole cell patch clamp recordings from ON cBCs in dark-adapted rabbit retinal slices superfused with Ames medium containing dopamine (5 μM) to mimic the effect of maintained (30 min) bright illumination, ON-cBCs (top row-control, DOPA CTL) exhibited both surround antagonism (i.e., the amplitude of center responses was reduced by simultaneous surround stimulation, as occurred in response to 500-μm (center and surround stimulation) vs. 50-μm (center stimulation only) wide bars, and surround activation (i.e., response to surround stimulation alone was opposite in polarity to the response produced by center stimulation alone, as occurred in response to near surround stimulation). In separate experiments, when the dopamine medium also contained the D_1_R antagonist SCH23390 (SCH; 5 μM) for >30 min (DOPA + SCH; second row from top) or when slices were superfused without dopamine (NO DOPA, third row from top) to mimic the effect of maintained darkness, ON-cBCs exhibited minimal surround antagonism and activation. When the dopamine medium also contained the D_2/3/4_R antagonist spiperone (SPI; 5 μM) for >30 min (DOPA + SPI; bottom traces), ON-cBCs exhibited both surround antagonism and activation. Surround responses were evoked by two simultaneously flashed bar stimuli equidistant from the receptive field center, i.e., having 500 μm (called 500 μm wide bar) or 50 μm (called 50 μm wide bar) distance between the bars. For near surround stimulation, the distance between 50-μm wide bars = 100 μm, and for far surround stimulation, distance between 100-μm wide bars = 500 μm. The dotted horizontal lines adjacent to the response traces denote the peak response amplitude of the cells to the smallest centered stimulus (50-μm wide bar). Modified from Chaffiol et al. (2017).

Cone bipolar cells, interneurons that receive visual input from cones and relay it to ganglion cells, have also been shown to exhibit receptive field surrounds with characteristics similar to those of ganglion cells (Werblin and Dowling, 1969; Dacey et al., 2000; Fahey and Burkhardt, 2003; Chaffiol et al., 2017). The center-surround receptive field characteristics of one type of cBC, ON-center cBCs (ON-cBCs; and ON-center ganglion cells), under three levels of maintained background illumination, are shown in Figures 1, 3. For each light level, the center-surround receptive field profile is shown in Figures 1A2–C2, center-surround light responses are depicted in Figure 3A, and chemical and electrical synaptic mechanisms that underlie changes in center and surround profiles and responses are shown in Figures 1A1–C1.

The surround light responses of ON-cBCs under maintained bright background illumination have two different inhibitory characteristics. As shown in Figures 1A2, 3A, surround stimulation in the presence of center stimulation (spot and annulus or ring of light) reduces the center response size of ON-cBCs, a phenomenon known as “surround antagonism” (Mangel, 1991; Thoreson and Mangel, 2012; Chaffiol et al., 2017) Also, ON-cBCs, which have depolarizing center light responses, produce hyperpolarizing responses to surround stimulation alone (only annulus of light is flashed) that are opposite in polarity to those produced by center (spot) stimulation alone. This kind of surround response is known as “surround activation” (Mangel, 1991; Chaffiol et al., 2017) Following maintained dim illumination, ON-cBCs exhibit surround antagonism but not surround activation. As can be seen by comparing Figures 1A2,B2, surround size and center size are both larger under dim illumination compared to bright illumination. It is worth noting that an important feature of the surround under maintained bright illumination is that in addition to being stronger (compared to under dim illumination), it is also smaller. This and its increased strength cause center size to also decrease, and the combination of smaller center and surround improves spatial discrimination and edge detection (see “Functional Considerations” section below). As also shown in Figures 1C1,C2 and 3A, following maintained darkness, ON-cBCs exhibit center responses but minimal or no surround (Thoreson and Mangel, 2012). However, despite observations over the course of many decades that surround light responses depend on the intensity of maintained background illumination (Barlow et al., 1957; Barlow and Levick, 1969; Werblin and Dowling, 1969; Hammond, 1975; Troy and Shou, 2002; Fahey and Burkhardt, 2003; Thoreson and Mangel, 2012), the mechanisms that underlie how light and dark adaptation modulate surround strength remained unclear.

**Figure 4:**
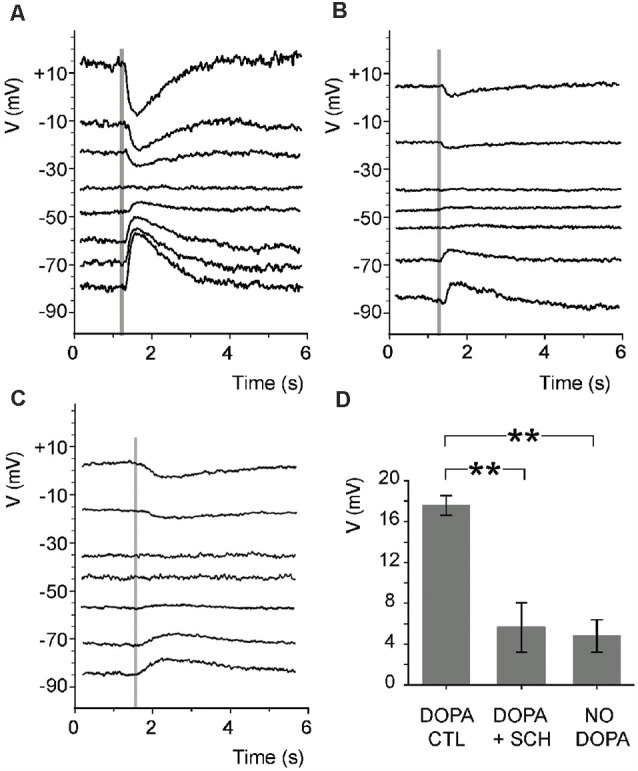
Endogenous activation of ON-cBC D_1_Rs leads to GABA_A_R activity of ON-cBC dendrites.In rabbit retinal slices superfused with Ames medium containing dopamine (5 μM), TPMPA (50 μM), a GABA_C_R antagonist, and cobalt (2 mM) to block synaptic transmission, GABA (0.5 mM) was puffed onto BC dendrites (time of puff is indicated by shaded vertical lines) by pressure ejection. GABA responses were clearly evident under dopamine (control) conditions **(A)** with an average GABA reversal *potential* (E_GABA_) = −42.2 + 2.6 (SEM) mV (*n* = 5), but were much reduced by SCH (5 μM) **(B)** and when the superfusate did not contain dopamine **(C)**. **(D)** When GABA was puffed at −70 mV, average peak GABA_A_R response size was significantly greater (**p < 0.01) when the superfusate contained dopamine (DOPA CTL; 17.6 + 1 mV; *n* = 5) compared to when it contained dopamine and SCH (DOPA + SCH; 5.6 + 2.4 mV; *n* = 5) or did not contain dopamine (NO DOPA; 4.8 + 1.6 mV; *n* = 5). Modified from Chaffiol et al. (2017).

A recent study has shown that the intensity of maintained background illumination modulates the surround light responses of rabbit ON-cBCs by regulating activation of dopamine D_1_Rs and GABA_A_Rs on the dendrites of the cells (Chaffiol et al., 2017). Dark-adapted retinal slices were bathed in dopamine to mimic the effect of maintained bright background illumination. In addition, because GABA_A_Rs are located on the dendrites of ON-cBCs (Greferath et al., 1994; Vardi and Sterling, 1994; Haverkamp et al., 2000; Shields et al., 2000) and GABA_A_R activation opens chloride channels, micropipettes that were used for whole-cell recording contained 23.7 mM Cl^−^, so that E_Cl_ = −42 mV (= average resting membrane potential of ON-cBCs at the start of recording). Under these conditions, ON-cBCs produced center and surround light responses, including surround activation and surround antagonism (Figure 3B). Blockade of D_1_Rs, but not blockade of D_2/3/4_Rs, eliminated both surround activation and antagonism. In addition, the cells in dark-adapted slices did not exhibit surround responses when the superfusion solution lacked dopamine. Other experiments demonstrated that when synaptic transmission was eliminated ON-cBCs exhibited GABA_A_R activity *(*i.e., ON-cBCs responded to direct GABA application) when dopamine was in the bath but not when D_1_Rs were blocked or when dopamine was not added to the bath (Figure 4). In addition, GABA_A_Rs were expressed on the dendrites-including dendritic tips-of ON-cBCs in the day following maintained bright illumination, but expressed minimally following maintained darkness or following maintained bright illumination after D_1_Rs were blocked for 30 min.

**Figure 5:**
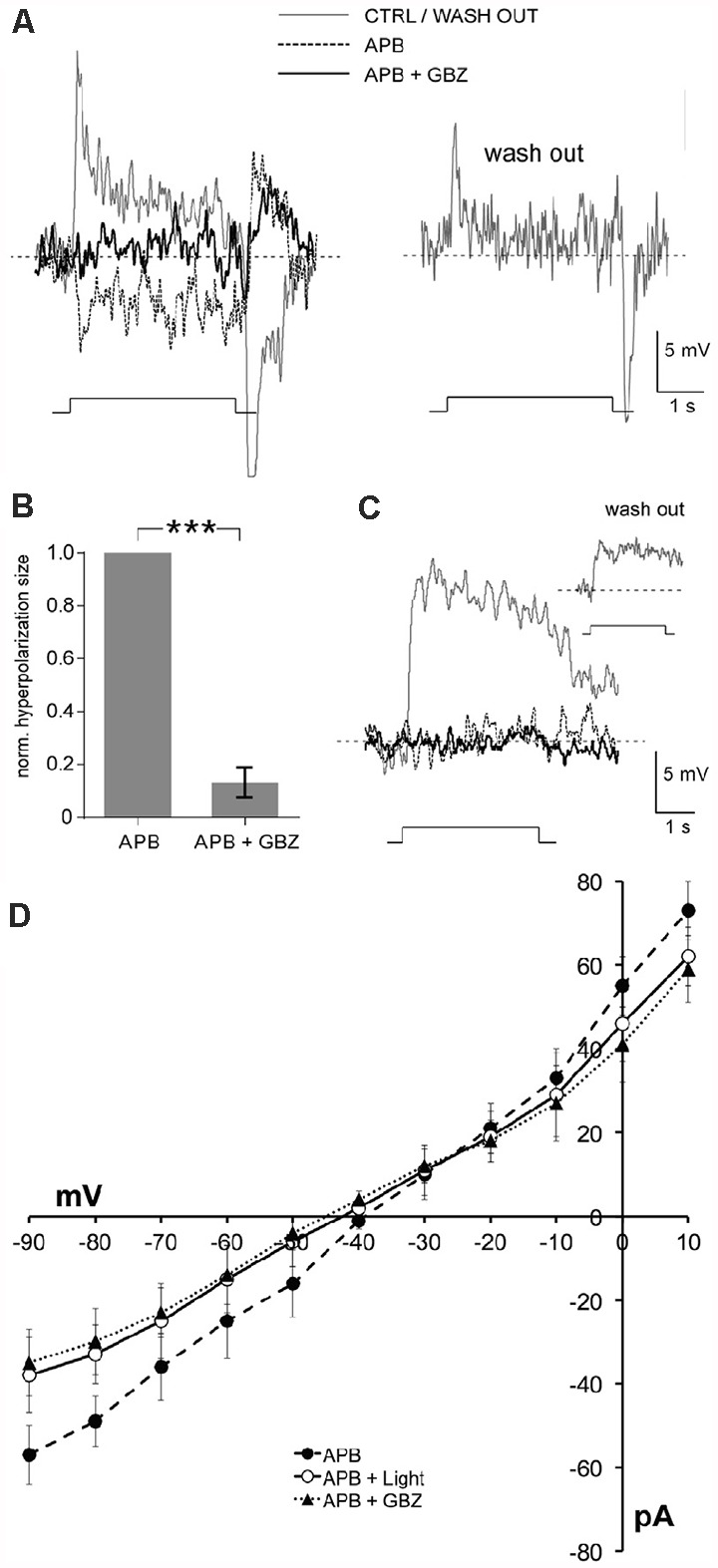
Hyperpolarizing surround light responses of ON-cBCs are produced by a reduction in tonic endogenous GABA_A_R excitation.In the presence of dopamine (5 μM) and the glutamate analog APB (i.e., L-AP4; 50 μM), a selective agonist of the mGluR6-Rs on both ON-cBC and rod BC dendrites that blocks cone to ON-cBC and rod to rod BC signaling (Slaughter and Miller, 1981), ON-cBCs (**A,B**), but not rod BCs **(C)**, produced hyperpolarizing surround responses to large (500-μm wide) centered bar stimuli that were blocked by gabazine (GBZ; 50 μM; 9 out of 9 cells). Washout of APB and GBZ showed recovery from their effects **(A–C)**. **(B)** For each ON-cBC, addition of GBZ to the APB-containing medium greatly reduced the average normalized size of hyperpolarizing surround light responses (***p < 0.001; paired *t-test*; *n* = 9). **(D)** Comparison of the current-voltage relationship of ON-cBCs (*n* = 5) following applications of APB alone in the dark and during surround light responses, and following application of both APB and GBZ in the dark. Average steady-state current at each holding potential (i.e., current measured near the end of the voltage pulses and when the amplitude of the light responses was relatively steady) is shown for all three experimental conditions. Modified from Chaffiol et al. (2017).

In addition, Chaffiol and colleagues (Chaffiol et al., 2017) showed that when D_1_Rs were activated and cone to ON-cBC transmission was blocked, the conductance of ON-cBCs was reduced by gabazine (GABA_A_R antagonist) and during hyperpolarizing surround light responses (Figure 5). These conductance measurements suggest that GABA tonically depolarizes ON-cBC dendrites following maintained D_1_R activation (i.e., maintained bright illumination) and that hyperpolarizing responses to surround stimulation can be attributed to reduced GABA_A_R excitation. It is worth noting that these results are inconsistent with the idea that surround stimulation evokes a GABA-mediated conductance that inhibits the cells (Chaffiol et al., 2017). The findings also suggest that the intracellular chloride concentration of ON-cBC dendrites following maintained bright illumination (when D_1_Rs are strongly activated) is such that E_GABA_ is more positive than the resting membrane potential, possibly due to activity of the chloride cotransporter NKCC on the dendrites of the cells (Vardi et al., 2000).

**Figure 6:**
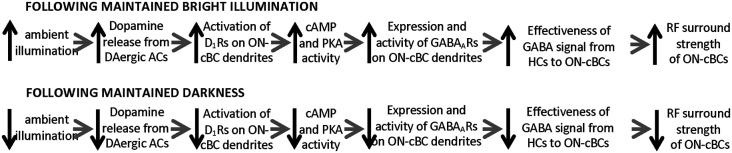
Model of how the ambient light level, by altering dopamine D_1_ receptor activation, modulates the GABA_A_ receptor-mediated receptive field surround of ON-cBCs.The model accounts for how changes in ambient (background) light level affect the GABA feedforward signal from HCs to ON-cBC dendrites that mediates the strength of ON-cBC surround responses . As ambient illumination slowly increases during the morning, reaching a peak at midday, D_1_R activation increases, which in turn augments intracellular PKA, so that the expression and activity of GABA_A_Rs on ON-cBC dendrites-including dendritic tips-are enhanced. As a result, the effectiveness of the GABA_A_R-mediated feedforward signal from HC dendrites to ON-cBC dendrites increases, enhancing the strength of surround antagonism and activation. Conversely, as background illumination slowly decreases during the afternoon and evening, reaching darkness at night, D_1_R activation is reduced. This in turn decreases intracellular PKA, substantially lowering GABA_A_R expression and activity so that ON-cBC surround strength is minimal.

Considered together, these findings demonstrated that light and dark adaptation modulate the surround light responses of ON-cBCs, as has been observed for ganglion cells in *in vivo* cat retina. Moreover, the results also suggest that maintained bright illumination increases ON-cBC dendritic D_1_R activation, which increases intracellular PKA, so that the expression and activity of GABA_A_Rs on ON-cBC dendrites are enhanced, producing ON-cBC surrounds (Figure 6). Conversely, maintained darkness decreases D_1_R activation, which decreases PKA, reducing GABA_A_R expression and activity and weakening ON-cBC surrounds (Figure 6). It is worth noting that although Chaffiol et al. (2017) showed that activation of D_1_Rs on ON-cBC dendrites increases GABA_A_R expression and activity and mediates surround activation and surround antagonism, the findings do not address the mechanisms by which maintained dim illumination produces surround antagonism but not surround activation. Moreover, in addition to the classic surround of cBCs and ganglion cells that is modulated by gradual changes in the maintained light level, other types of surround with different response characteristics are produced in the inner retina (Thoreson and Mangel, 2012).

### Dopamine-Mediated Light-Dark Modulation of HC Coupling

Current injections into HCs have directly demonstrated the contribution of HCs to BC surround antagonism and activation in the following ways: (1) changing the membrane potential of HCs modulates the membrane potential of nearby BCs and the spiking of nearby ganglion cells; and (2) artificially hyperpolarizing HCs, as occurs when they respond to light stimuli, antagonizes the center light responses of nearby BCs and ganglion cells (Naka and Nye, 1971; Toyoda and Kujiraoka, 1982; Mangel and Miller, 1987; Mangel, 1991; Mangel and Brunken, 1992).

Although it is accepted that cBC dendrites express GABA_A_Rs and respond to exogenous GABA (Wässle et al., 1998; Shields et al., 2000), evidence that HC dendrites express synaptic vesicles, the transmitter GABA, and synaptic machinery to release GABA when the dendrites are depolarized has been difficult to obtain. However, as discussed in detail (Thoreson and Mangel, 2012), recent observations suggest that a GABA signal from HCs activates GABA_A_Rs on cBC dendrites: (1) HCs express GABA (Deniz et al., 2011); (2) HCs have synaptic machinery to release GABA when depolarized (Guo et al., 2009, 2010; Hirano et al., 2011); and (3) the close spatial proximity of GABA_A_R subunits on cBC dendrites (Vardi and Sterling, 1994) to the vesicular GABA transporter on HC dendrites (Haverkamp et al., 2000), a likely GABA release site, suggests that HCs release GABA to activate GABA_A_Rs on cBC dendrites. Moreover, the conductance measurements of ON-cBCs described above (see Figure 5) show that during D_1_R activation, the conductance of ON-cBCs was reduced by gabazine (GABA_A_R antagonist) and during hyperpolarizing surround light responses (Chaffiol et al., 2017). These results suggest that GABA tonically released from HCs depolarizes ON-cBC dendrites following maintained D_1_R activation (i.e., maintained bright illumination) and that hyperpolarizing surround responses can be attributed to reduced GABA_A_R excitation. Moreover, it is well documented that bright light uncouples HCs by activating their D_1_Rs (Dowling, 2012), thereby reducing the size of their receptive fields (Thoreson and Mangel, 2012). Thus, all together the evidence suggests that HCs use GABA to provide a surround signal to cBCs and that the effectiveness of the HC signal depends on the expression and activity of GABA_A_Rs on the dendrites of ON-cBCs (Figure 6). In addition, the size of the ON-cBC surround is dependent on the extent of HC coupling (Figure 1), which itself is dependent on the background light level and the extent of D_1_R activation. Thereore, as increases in maintained illumination during the morning enhance ON-cBC surround strength, the size of cBC center and surround and the size of HC receptive fields decrease (Chaffiol et al., 2017). These light-adaptive processes improve detection of edges and small spatial details. Conversely, as maintained illumination gradually decreases during the afternoon, cBC surround strength decreases, and the size of cBC center and surround and the size of HC receptive fields increase (Figures 1, 6; Thoreson and Mangel, 2012; Chaffiol et al., 2017).

Recent work has suggested that D_1_Rs are expressed by most mouse cBCs, but not by all cBC subtypes (Farshi et al., 2016) and that D_1_R effects may be cBC subtype-specific (Hellmer et al., 2020). These results raise the interesting possibility that one important effect of D_1_Rs is to decorrelate the activity of different cBC subtypes, implying that ganglion cell subtypes and their post-synaptic targets through the visual system (Caldwell and Daw, 1978; Mangel et al., 1983; Troy and Shou, 2002; Wienbar and Schwartz, 2017) may be differentially affected as well. Further work is needed in this area of research.

## Dopamine-Mediated Intrinsic Modulatory Processes in The Outer Retina

### Circadian Clock Pathways

**Figure 7:**
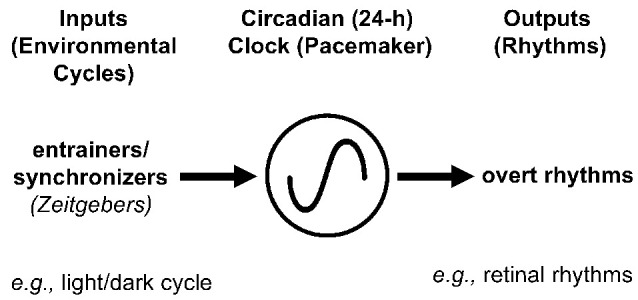
Simplified circadian clock pathway in the retina. A simplified circadian clock pathway has three components. First, there is a molecular mechanism, which is referred to as a clock, clockwork, or a pacemaker, that has a period of approximately 24 h and is able to maintain its daily rhythmicity in the absence of environmental cues (e.g., in constant darkness and temperature). Second, although circadian clocks can maintain rhythmicity in a constant environment, they are entrained or synchronized to the local environment by zeitgebers (German for time-giver). For example, the daily light/dark cycle acts as an input to clocks because the onset of morning light resets the “hands” of clocks so that they are set to local time, even though environments do not alter the period of clocks. Third, circadian clocks produce daily rhythms at the molecular, cellular, and systems levels. For example, the circadian clock in the retina produces a variety of daily rhythms within the retina, including increasing the hormone melatonin at night, decreasing the neurotransmitter dopamine in the day, and enabling cone photoreceptors to respond to very dim light stimuli at night (but not in the day). Because circadian clocks are affected by environmental stimuli, circadian experiments, which aim to determine whether time of day or night affects a function (e.g., level of extracellular dopamine), are conducted in the absence of environmental cues (e.g., under conditions of constant darkness and temperature). In a circadian experiment, the terms “subjective day” and “subjective night (SN)” refer to the day and night of the imposed light/dark cycle, respectively, when animals or isolated intact retinas were maintained in constant darkness.

A simplified version of a circadian clock pathway that consists of a circadian clock, inputs to it such as the light/dark cycle, and clock outputs, which are referred to as circadian rhythms, is shown in Figure 7. We include this figure for readers new to the field of circadian biology but it is important to remember that all three components of a retinal circadian clock pathway are far more complex than is depicted here. Readers interested in the molecular components (or clockwork) of the retinal circadian clock, input pathways to the retinal clock, or diverse effects of the retinal clock not discussed here should refer to reviews that explore these topics in detail (Iuvone et al., 2005; Besharse and McMahon, 2016; Ko, 2018). This section will focus on the role of dopamine as an effector of the intrinsic retinal circadian in one of its output pathways.

### Roles of Melatonin and Dopamine as Effectors of the Retinal Circadian Clock

**Figure 8:**
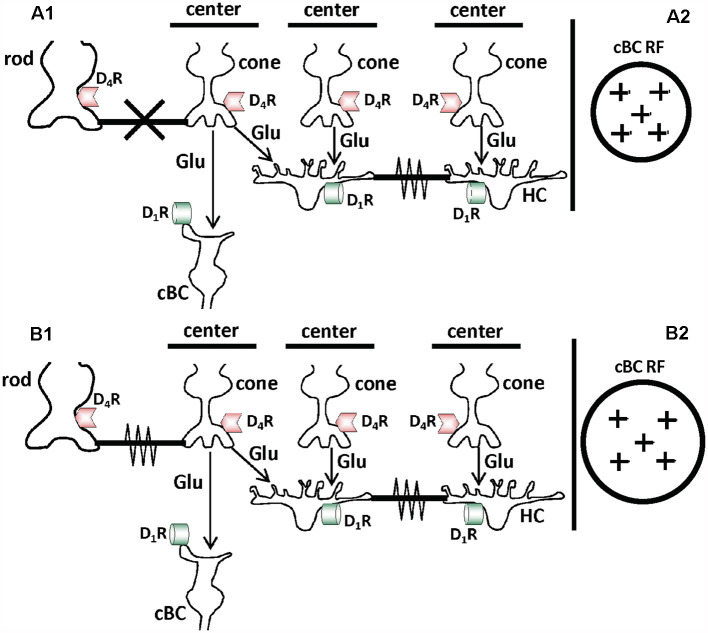
Schematic diagrams showing that the retinal circadian clock, by regulating activation of D_4_ receptors on cones and rods, controls electrical synaptic signaling between rods and cones and the receptive field size of cones. **(A1,B1)** Cell types, transmitter receptors, and gap junctions are the same as in Figure 1, but the retinal clock regulates their expression and function in day and night differently compared to the effects of background illumination. The different effects of the retinal clock and background illumination occur because the circadian clock and light activate D_4_Rs and D_1_Rs, respectively (Figure 2). Although the retinal clock releases less dopamine in the day than bright light, circadian clock-induced dopamine release in the day is sufficient to activate cone D_4_Rs (but not D_1_Rs; Figure 2). This is because D_4_Rs are ~500 times more sensitive to dopamine than D_1_Rs. In constant darkness at night, dopamine levels are lower than in the day and not sufficient to activate cone D_4_Rs. The retinal clock, therefore, controls D_4_R-mediated phenomena such as rod-cone coupling but not D_1_R-mediated effects such as GABA_A_R expression and function on cBC dendrites and gap junction coupling between HCs. As a result, in maintained darkness in both day and night when D_1_Rs are not activated, cBC dendrites lack GABA_A_Rs, HC gap junctions are open, and cBCs lack receptive field surrounds. **(A1,A2)** Following maintained darkness in the day, the retinal clock releases sufficient dopamine to activate D_4_Rs in cones and rods, which decreases cAMP/PKA in the cells. As a result, gap junctions between rods and cones are closed **(A1)**. The RF center of cBCs is large because they lack RF surrounds due to the lack of GABA_A_Rs on its dendrites. However, the RF center of cBCs may not be as large as at night **(A2)**. **(B1,B2)** Following maintained darkness at night, the retinal clock does not release enough dopamine to activate D_4_Rs in cones and rods, which increases cAMP/PKA in the cells. As a result, gap junctions between rods and cones are open **(B1)**. Evidence indicates that the RFs of cones are larger at night in the dark than in the day in the dark due to increased photoreceptor coupling. It is hypothesized that the RF center of cBCs is therefore larger at night in the dark than in the day **(B2)**.

Figures 8A,B depict the same cell types, transmitters, transmitter receptors, and chemical and electrical synapses as shown in Figure 1. However, Figures 8A,B show that the gap junctions between rods and cones are closed in the day in the dark but open at night in the dark due to the action of the retinal clock. In addition, although the receptive field center of cBCs is large in the day in the dark (Figure 8A), it is hypothesized to become even larger at night (Figure 8B) due to the increase in rod-cone coupling and the resultant increase in the size of cone receptive fields (Ribelayga et al., 2008). No other day/night changes are shown in Figures 8A1,B1, i.e., the retinal clock does not affect HC coupling or GABA_A_R expression and activity on the dendrites of ON-cBCs. These processes are not affected by the retinal clock because the clock-elicited increase in dopamine release in the day is not sufficient to activate low-affinity D_1_Rs, which control HC coupling and GABA_A_R function on ON-cBC dendrites (see Figures 1–3).

**Figure 9:**
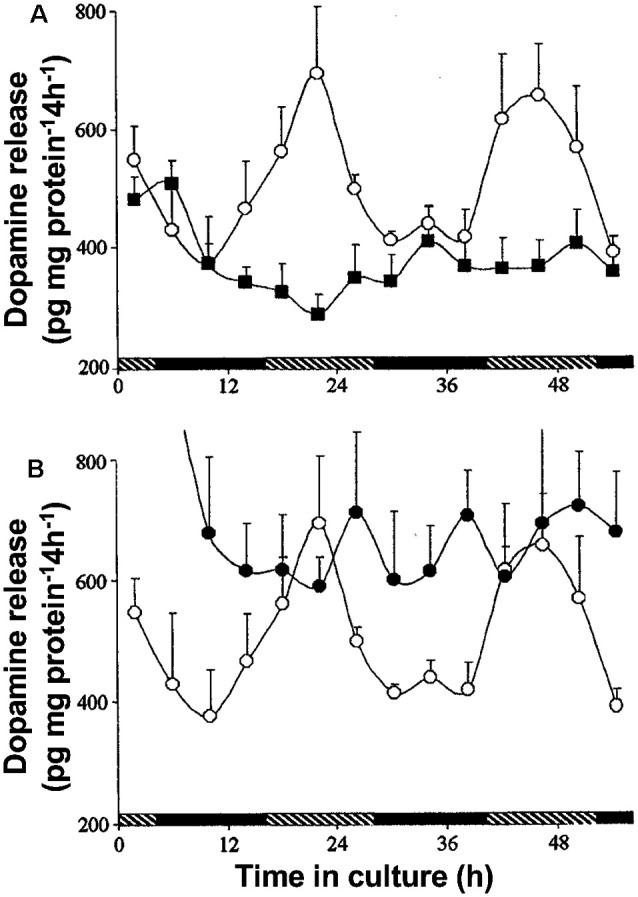
The retinal clock uses melatonin to produce the circadian release of dopamine. **(A,B)** Isolated intact goldfish neural retinas were maintained for 56 h in a culture medium in constant darkness and temperature. High performance liquid chromatography was used to measure dopamine levels in culture medium sampled every 4 h. A clear circadian rhythm was observed with higher levels of dopamine during the day. **(A)** Continuous application of melatonin (1 nM; filled squares) to retinas for 56 h, abolished the rhythm of dopamine release by decreasing daytime levels to nighttime values. **(B)** Continuous application of the selective melatonin antagonist luzindole (1 μM; filled circles) abolished the rhythm of dopamine release by increasing the nighttime values to daytime levels. Each data point represents mean values ± SEM. Open circles in both **(A)** and **(B)** represent positive controls performed at the same time, but with no test drugs added. Hatched and filled bars indicate the subjective day and night, respectively. Modified from Ribelayga et al. (2004).

The retinal circadian clock does not directly control dopamine levels in the retina. Rather, it affects dopamine through its action on the hormone melatonin. Dopamine release from the retina exhibits a circadian rhythm in which it is higher in the day than at night (Figure 9 here; also see Doyle et al., 2002b; Ribelayga et al., 2004). Melatonin has been shown to also have a circadian rhythm (Mangel and Ribelayga, 2010; Besharse and McMahon, 2016; Ko, 2018), but it is higher at night than in the day. The retinal circadian clock controls the synthesis of melatonin by controlling the activity of N-acetyl transferase (NAT), the rate-limiting enzyme in melatonin production. Because melatonin inhibits dopamine release in the retina (Dubocovich, 1983; Reppert et al., 1995), the melatonin rhythm produces a dopamine rhythm that is opposite in phase, i.e., when melatonin is high at night, dopamine is lower, and when melatonin is lower in the day, dopamine is higher. This relationship between the rhythms of melatonin and dopamine is shown by the experiments depicted in Figure 9. When goldfish retinas were maintained for ~52 h in constant darkness and temperature, a rhythm of dopamine was observed in the cultured medium that was sampled every 4 h (Figure 9A). However, when melatonin was continuously applied, it abolished the rhythm in dopamine release by suppressing it in day to the low level typical of the night (Figure 9A). Conversely, continuous application of the melatonin receptor antagonist, luzindole, abolished the rhythm in dopamine release by increasing dopamine release at night to its typically higher level in the day (Figure 9B). Although it has been reported that dopamine reciprocally inhibits melatonin (Cahill and Besharse, 1991; Hasegawa and Cahill, 1999; Tosini and Dirden, 2000; Doyle et al., 2002a), the overall interaction between melatonin and dopamine is that endogenous melatonin inhibits dopamine release and produces the daily rhythm in dopamine release. Therefore, dopamine acts as a circadian clock signal for the day, while melatonin acts as a circadian signal for the night.

### Dopamine D_4_R-Mediated Regulation of Rod Input to Cone Horizontal Cells

**Figure 10:**
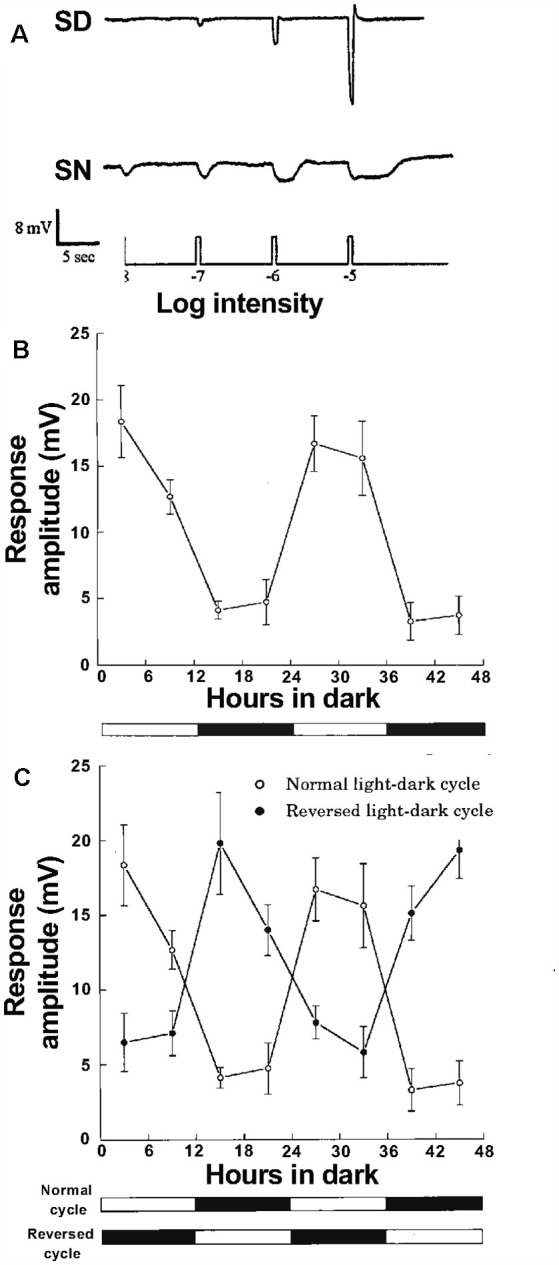
Cone horizontal cells respond to very dim (low scotopic) light stimuli at night but not in the day due to action of a circadian clock. **(A)** Responses of L-type cone horizontal (H1) cells to dim full-field white light flashes (ranging from –8 log*I*_o_ to –5 log*I*_o_) in dark adapted retinas show that cone input to H1 cells dominates during subjective day (SD) and rod input dominates during subjective night (SN). Compared to the day, the responses at night are slower, smaller in size, longer in duration, and response threshold is approximately two log units lower. **(B)** Average stimulus intensity that generated a threshold (1 mV) response from H1 cells as a function of time in the dark was lower in the subjective night than in the subjective day, indicating that a circadian clock regulates rod input to the cells. **(C)** Following reversal of the light/dark cycle for 10 days before each experiment, average threshold responses reversed as well, i.e., threshold was approximately two log units lower in the subjective night (which before reversal was the daytime) than in the subjective day. **(A–C)** Cells were recorded with intracellular micropipettes. Impalement was achieved in complete darkness, except for the use of flashing dim (< −6 log*I*_o_) lights to confirm that impalement had occurred. Once HC recording began, the threshold light response was determined by flashing full-field white lights (ranging from –9 log *I*_o_ to –5 log *I*_o_; 500 ms duration at 0.1 Hz) in half log unit steps. Control experiments have determined that the dark-adapted state of the retina is maintained at night as long as no stimuli brighter than −4.5 log*I*_o_ (low mesopic) are flashed even once (Wang and Mangel, 1996; Ribelayga et al., 2008). **(A–C)** The maximum, unattenuated intensity (*I*_o_) of full field white light stimuli from a 100-W tungsten-halogen lamp was 5.0 × 10^3^ W•cm^−2^. Intensity values indicated in the text are relative to *I*_o_. **(A)** Modified from Ribelayga et al. (2002). **(B,C)** Modified from Wang and Mangel (1996).

Diverse cellular and molecular processes in the vertebrate retina display circadian rhythms and many of these rhythms are regulated by the circadian clock in the retina (Mangel, 2001; Iuvone et al., 2005; Mangel and Ribelayga, 2010; Besharse and McMahon, 2016; Ko, 2018). Some early studies had reported that cone-connected HCs could respond to dim light stimuli (low scotopic) following maintained dark adaptation (e.g., cat: Steinberg, 1969; goldfish: Mangel et al., 1994), but the mechanism underlying their sensitivity to scotopic stimuli remained unclear. “Scotopic” refers to the range of dim light intensities in which rods, but not cones, initiate the visual process. The first report at the single neuron level that the retinal clock affects the light responses of individual neurons in the vertebrate retina was the finding that the light responses of cone horizontal cells (cHCs), which make synaptic contact with cones, but not with rods (Stell and Lightfoot, 1975), exhibit a day/night difference in constant darkness (Wang and Mangel, 1996). As shown in Figures 10A,B, goldfish cHCs respond to very dim light stimuli (low scotopic) at night in the dark but not in the day in the dark (Wang and Mangel, 1996; Ribelayga et al., 2002). In this series of experiments, an important control was performed to demonstrate that this daily difference in sensitivity to dim light stimuli is controlled by a circadian clock. Specifically, even though the above experiments were performed in constant darkness, it is possible that the observed day/night difference was not due to a circadian clock but to a day/night difference in an environmental factor, such as a decrease in temperature at night. This possibility was ruled out by showing that prior reversal of the light/dark cycle for 10 days reversed the circadian rhythm in sensitivity (Figure 10C; Wang and Mangel, 1996), i.e., after light/dark cycle reversal. peak sensitivity occurred during what used to be the day (even though other factors such as daily temperature changes had not been reversed). Spectral sensitivity measurements also showed that a specific type of goldfish cHC, the L-type (H1) cHC, which receives synaptic contact primarily from red cones (Stell and Lightfoot, 1975), had a spectral sensitivity similar to that of goldfish rods at night, but similar to that of red cones in the day (Wang and Mangel, 1996). These findings thus strongly suggested that rod input reaches cHCs at night but not in the day.

Because isolated cones require absorption of 100–1,000 times more photons compared to rods to produce small light responses (Dowling, 2012), the finding that cHCs in intact retina had sensitivity to very dim light stimuli similar to that of rods was striking. Because goldfish cHCs do not make synaptic contact with rods (Stell and Lightfoot, 1975), we hypothesized that rod input reaches cHCs at night *via* open rod-cone gap junctions and that rod input does not reach cHCs in the day because the gap junctions close (Wang and Mangel, 1996). This idea was strengthened by subsequent studies on goldfish cHCs (Ribelayga et al., 2002, 2004) that showed that the circadian rhythms in light responses, spectral sensitivity, and dim light sensitivity depended on dopamine D_2-like_ receptors (i.e., D_4_Rs), which are on cones but not HCs (Witkovsky, 2004). If the hypothesis that rod input reaches cHCs at night via open gap junctions is correct, then cones should respond to dim light at night like cHCs.

### Dopamine D_4_R-Mediated Regulation of Rod-Cone Gap Junction Coupling

**Figure 11:**
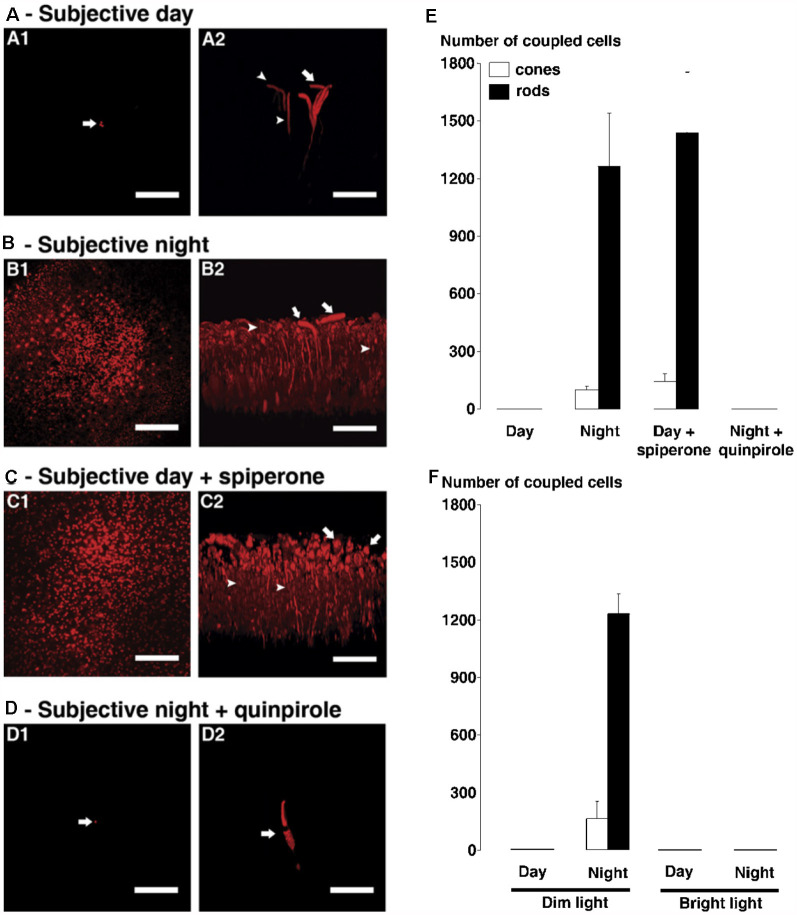
The circadian clock in the goldfish retina controls rod-cone tracer coupling by activating dopamine D_4_ receptors in the day but not at night. **(A–D)** Following iontophoresis of biocytin into individual cones in intact neural goldfish retina, tracer remained in a few cells (indicated by arrows) near the injected cone during the subjective day **(A)** and during the subjective night in presence of D_4_ receptor agonist quinpirole (1 μM, **D**). It diffused into many rods and cones during subjective night **(B)** and during subjective day in the presence of D_4_ receptor antagonist spiperone (10 μM, **C**). In each of **(A–D)**, confocal images of a whole-mount retina at the level of the rod inner segments are shown on the left **(A1–D1)** and perpendicular views of the 3-D reconstruction of the photoreceptor cells from the same retina are shown on the right **(A2–D2)**. Some cones and rods are indicated by arrows and arrowheads, respectively. Scale bars **(A–D)**: 50 μm. **(E,F)** Average numbers of stained cones (open bars) and rods (filled bars) following biocytin injections into individual cones (1 cone injected/retina) under dark-adapted **(E)** and light-adapted **(F)** conditions are shown. **(E)** Under dark-adapted conditions (>60 min), the number of tracer coupled rods and cones was significantly greater during the night and during the day following spiperone treatment than during the day under control conditions. **(F)** Under dim light-adapted conditions (i.e., −5 log*I*_o_, 500 ms light flashes at 0.125 Hz for >60 min), the number of tracer-coupled rods and cones was significantly greater during the night compared to the day (i.e., results are similar to those obtained in the dark). In contrast, under bright light-adapted conditions (i.e., −2 log*I*_o_, 500 ms light flashes at 0.125 Hz for >60 min) in day and night, biocytin was restricted to the injected cone; no other cells were labeled. Error bars represent SEM. Modified from Ribelayga et al. (2008).

Rod-cone gap junctions have been observed in diverse vertebrate species such as fish, amphibia, and mammals including primates (Raviola and Gilula, 1973; Bloomfield and Völgyi, 2009). Injections of tracer into individual goldfish cones in day and night under dark- adapted conditions with or without spiperone (selective D2R family antagonist) or quinpirole (selective D2R family agonist) have confirmed the hypothesis that the retinal circadian clock controls rod-cone coupling through activation of cone D_4_Rs (Ribelayga et al., 2008). Specifically, tracer diffused into an average of 1,200 rods and 100 cones during the subjective night (SN) and during the subjective day (SD) in the presence of spiperone but was restricted to an average of two cones and two rods during the subjective day and during the subjective night in the presence of quinpirole (Figure 11). In addition, use of a technique (Choi et al., 2012) called “cut loading” in which a razor blade dipped in neurobiotin tracer cut through goldfish and mouse retinal tissue showed that tracer had diffused through photoreceptors further from the cut at night in the dark than in the day in the dark (Ribelayga et al., 2008).

Interestingly, similar results were obtained when low-mesopic light stimuli, such as occur naturally right before dawn or just after dusk, were flashed onto the retinas for >60 min before tracer injections (Figure 11F). This result strongly suggests that the retinal clock, and not the retinal response to the normal visual environment at night, controls rod-cone coupling. Note that brighter light such as occurs typically during the day (but not naturally at night) closed rod-cone gap junctions in both day and night (Figure 11F). It is common for bright illumination to block circadian clock effects, a phenomenon known as “masking” (Ribelayga et al., 2008).

**Figure 12:**
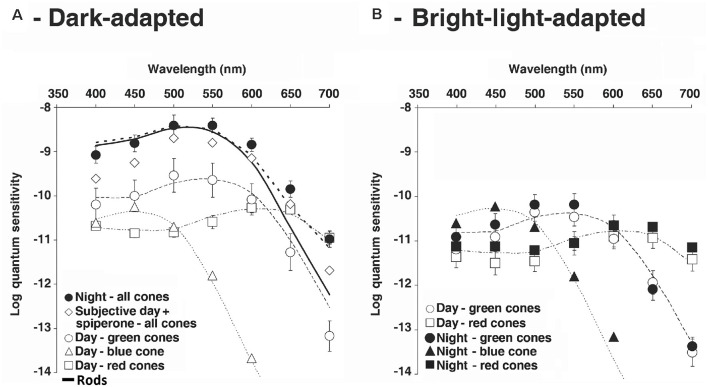
Following maintained darkness goldfish cones exhibit red, green or blue spectral sensitivity in the day, but at night spectral sensitivity is similar to that of rods. **(A,B)** Cone spectral sensitivity resembled that of rod during the night in the dark and in the day following spiperone application, but not in the day in the dark or in the light. These results demonstrate that rod-cone gap junctions are function all open at night in the dark and in the day following blockade of D_4_Rs, functionally closed in the day. **(A)** Average spectral sensitivity of cones recorded under dark-adapted conditions during the day or subjective day fit one of three nomograms (thin dotted curves) corresponding to the three major known types of goldfish cone pigments: L, M, and S. Data were obtained from recorded red cones (open squares), green cones (open circles) and blue cone (open triangles). In contrast, the spectral sensitivity of all dark-adapted cones recorded at night peaked at ~535 nm (filled circles). The cone spectral sensitivity at night under dark-adapted conditions closely fit a modified nomogram that combines goldfish rod and L-cone pigment nomograms (dotted thick curve; λ_max_ = 537 ± 3 (SD) nm; *r*^2^ = 0.91). Following application of spiperone (10 μM; open diamonds), cone spectral sensitivity in the subjective day resembled that observed during the subjective night and data points fit well the modified nomogram (λ_max_ = 537 ± 3 nm; *r*^2^ = 0.96). **(B)** Following bright light adaptation at night or during the subjective nightthree groups of cones with different spectral sensitivities were observed: red cones (filled squares), green cones (filled circles) and blue cone (filled triangles), whereas bright light adaptation during the day or subjective day did not affect the relative spectral sensitivity of the cones (red cones: open squares; green cones: open circles) but slightly decreased the absolute sensitivity. Nomograms as in **(A).** Data points represent average sensitivity ± SEM. Modified from Ribelayga et al. (2008).

**Figure 13:**
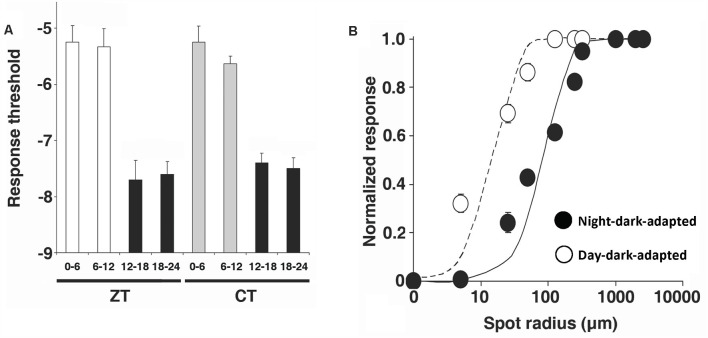
Retinal circadian clock increases receptive field size and sensitivity of cones to dim light by enhancing rod-cone coupling at night. **(A)** Under dark-adapted conditions, the average cone light response threshold (log intensity required to elicit a 0.5 mV response) was significantly higher during the day and subjective day than during the night and subjective night. **(B)** Average normalized response amplitudes of dark-adapted cones plotted against stimulus radius for a stimulus of intensity -5 log*I*_o_, indicates that the receptive field size of cones is larger at night (filled circles) than in the day (open circles). Error bars indicate SEM. Modified from Ribelayga et al. (2008).

Patch-clamp recordings from goldfish cones yielded results similar to those obtained from goldfish cHCs, i.e., cone spectral sensitivity was similar to that of rods during the subjective night and during the subjective day in the presence of spiperone, but during the day in the dark or following bright light adaptation green-sensitive, red-sensitive, and blue-sensitive cone spectra were recorded (Figure 12). These spectral sensitivity data demonstrate that rod-cone gap junctions are functionally open at night in the dark. In addition, patch-clamp recordings showed that cones were able to respond to very dim (low scotopic) light stimuli at night in the dark but not in the day (Figure 13A). It is worth noting that application of spiperone had no effect on cones at night in the dark (Ribelayga et al., 2008), as reported previously for cHCs (Ribelayga et al., 2002), indicating that the retinal clock decreases extracellular dopamine at night below the threshold of D_4_R activation (Figure 2). In addition, measurements indicated that the receptive fieldsize of cones was larger at night in the dark compared to the day in the dark (Figure 13B), consistent with the finding that rod-cone electrical synapses are open at night.

It is worth noting that a recent study of day/night differences in cone to cHC synaptic transmission in goldfish retina reported that although changes in rod-cone coupling can account for some day/night changes, such as changes in spectral tuning and response threshold of cones and cHCs, other day/night differences may result from distinct clock effects (Ribelayga and Mangel, 2019). For example, at night compared to the day cone to cHC synaptic transfer was highly non-linear and of lower gain. As a result, cHC light responses saturated at a lower intensity at night than in the day, and at a lower intensity than cones at night. These characteristics restrict cone to cHC signaling to very dim light stimuli, making the cone to cHC synapse more sensitive to small changes in dim light intensity at night (Ribelayga and Mangel, 2019).

**Figure 14:**
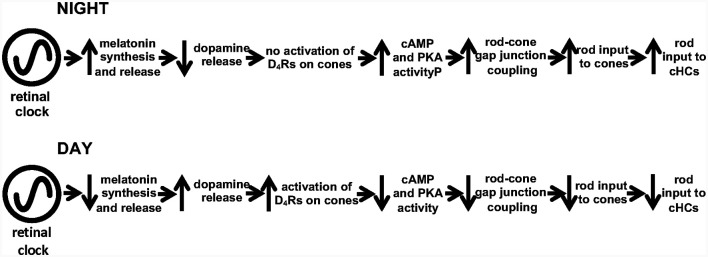
Summary of how the retinal circadian clock uses D_4_ receptors to control rod-cone gap junction coupling. During the subjective night, the circadian clock decreases dopamine release from dopaminergic neurons so that cone D_4_Rs are NOT activated, which increases intracellular cAMP/PKA. This increases rod-cone coupling (i.e., increases the conductance of rod-cone gap junctions) and the amount of rod input that reaches cones and cHCs. Conversely, during subjective day, the circadian clock in the retina increases dopamine release from dopaminergic neurons, activating D_4_Rs on rods and cones. This in turn decreases cAMP/PKA, so that the conductance of rod-cone gap junctions and the amount of very dim light rod signals that reaches cones and cHCs are decreased.

Considered together, these results demonstrate that the retinal clock increases dopamine release in the day by decreasing melatonin production in the day. Dopamine released from dopaminergic interplexiform cells (goldfish) or dopaminergic amacrine cells (mouse, rabbit) activates cone D_4_Rs, which decreases cAMP/PKA and closes rod-cone gap junctions (Figure 14). In contrast, during the subjective night, the retinal clock increases melatonin release, which reduces dopamine release. As a result, cone D_4_Rs are not activated. This results in an increase in intracellular cAMP/PKA, which opens rod-cone gap junctions so that rod input reaches cones and then cHCs (Figure 14).

It is important to emphasize that cones (and cHCs) are able to respond to very dim scotopic light stimuli at night in the dark due to the action of the retinal clock. In other words, cones and post-synaptic neurons in cone pathways respond to very dim (low scotopic) light stimuli at night because the conductance of the rod-cone electrical synapse is high. This phenomenon was observed by maintaining retinas under dark-adapted conditions with no illumination present brighter than low mesopic (i.e., not even a single brief flash; Wang and Mangel, 1996; Ribelayga et al., 2002, 2004, 2008; Ribelayga and Mangel, 2003, 2010). “Mesopic” refers to the range of light intensities in which both rods and cones initiate the visual process. It was assumed for years that rod-cone gap junctions open when background illumination reaches the mesopic range in the morning. This idea was based in part on the observation by many that bright light stimulation of dark-adapted retinas introduces a “rod plateau potential” or “depolarizing afterpotential” into cone and cHC light responses by slightly increasing rod-cone coupling (e.g., Yang and Wu, 1989; Krizaj et al., 1998; Witkovsky, 2004). However, as has been shown (Figures 5A,B in Ribelayga et al., 2008), rod plateau potentials in response to bright light stimulation occur when retinas are previously dark-adapted but not when retinas are previously light-adapted. Moreover, rod plateau potentials that occurred following dark adaptation were eliminated following 5 min of bright light stimulation (Figure 5B in Ribelayga et al., 2008). In addition, rod plateau potentials occur when retinas are dark adapted in the late afternoon or evening (when the retinal clock has begun to slowly open rod-cone gap junctions), but not when retinas are previously dark-adapted in the morning or at midday (Mangel, unpublished observations). Considered together, these results show that the presence of rod plateau potentials in cone (and cHC) responses to bright light stimulation following dark adaptation depends on the time of day and does not indicate that rod-cone coupling has been increased by bright illumination. Rather, the results suggest that rod plateau potentials in response to bright light stimulation following dark adaptation in the afternoon or evening reveal that rod-cone gap junctions are slightly open due to action of the retinal clock. In fact, bright light stimulation over the course of ~5 min eliminates rod plateau potentials of cones and cHCs by closing rod-cone gap junctions (Wang and Mangel, 1996; Ribelayga et al., 2008).

### Cone Dopamine D_4_ and Adenosine A_2A_ Receptors Work Together to Produce a Large Day/Night Difference in Rod-Cone Coupling

In addition to the melatonin/dopamine system, the purine adenosine also acts as an effector of the circadian clock in the retina (Cao et al., 2021). Adenosine, which is converted enzymatically from extracellular ATP that has been synaptically released with other transmitters (Ribelayga and Mangel, 2005; Cao et al., 2021), has an opposite phase compared to dopamine (Ribelayga and Mangel, 2005; Cao et al., 2021). In contrast to dopamine, extracellular adenosine levels increase during the night and as more adenosine binds to A_2A_ receptors (A_2A_Rs; a G_s_ protein receptor) on photoreceptors, there is downstream activation of cAMP and PKA and hence increased rod-cone coupling (Li et al., 2013; Cao et al., 2021). Because dopamine levels fall below the threshold of D_4_R activation at night, the large increases in intracellular cAMP/PKA and rod-cone coupling are not due simply to the lack of D_4_R activation. Rather, endogenous activation of A_2A_ receptors plays an important role in producing a large day/night difference in rod-cone coupling (Cao et al., 2021).

The circadian clock-induced increase in extracellular adenosine at night in the dark occurs in concert with an increase in intracellular adenosine (Ribelayga and Mangel, 2005; Cao et al., 2021). Because a circadian clock increases energy metabolism in both fish (Dmitriev and Mangel, 2000, 2004) and rabbit retina (Dmitriev and Mangel, 2001), it is likely that the clock-induced increase in the level of intracellular adenosine at night is due to a circadian-induced increase in energy metabolism. An attractive hypothesis is that neural activity and oxygen consumption may increase at night due to the action of the clock so that a slightly hypoxic condition is generated, thereby triggering the intracellular accumulation of AMP, a substrate for adenosine. In support of this, anoxic and hypoxic experimental conditions increase adenosine content and overflow in rabbit retinas (Ribelayga and Mangel, 2005). Evidence also suggests that the increase in extracellular adenosine at night in the dark occurs because the increase in intracellular adenosine at night is sufficient to stop the uptake of adenosine (Ribelayga and Mangel, 2005; Cao et al., 2021).

Both adenosine and dopamine levels are regulated by a circadian clock in the goldfish retina itself (Ribelayga et al., 2004; Cao et al., 2021). However, adenosine levels are not altered by short-term (1–3 h) application of dopamine and melatonin receptor agonists and antagonists (Cao et al., 2021), suggesting that adenosine levels in the day and night are independent of melatonin and dopamine receptors. Both the cone A_2A_Rs and D_4_Rs, which have opposite effects on intracellular adenylyl cyclase/PKA activity, might modulate rod-cone coupling *via* a simple additive effect on PKA activity at all times of day and night. Alternatively, cone A_2A_Rs and D_4_Rs might interact in their modulatory effects on rod-cone coupling (Li et al., 2013). For example, cone D_4_Rs signal through adenylyl cyclase 1 (Jackson et al., 2009), an isoform of adenylyl cyclase that is stimulated by Ca^2 +^-calmodulin and very sensitive to intracellular Ca^2 +^. Conversely, adenylyl cyclase 1 is poorly activated by Gs protein linked receptors (Sadana and Dessauer, 2009) such as A_2A_Rs, which inhibit Ca^2 +^influx into cone synaptic terminals (Stella et al., 2007). Because light adaptation produces a large decrease in intracellular Ca^2 +^ in cone synaptic terminals (Johnson et al., 2007), the increase in ambient illumination at dawn may augment the inhibitory effect of Gi on adenylyl cyclase 1. Moreover, this interactive process may run in reverse at dusk. Such an interaction might speed up transitions to cone vision at dawn and rod vision at dusk as coupling is decreasing and increasing, respectively. It has also been suggested that rhythmic expression of the genes for D_4_Rs and A_2A_Rs contributes to the regulation of rod-cone coupling at dawn and dusk (Li et al., 2013).

## Functional Considerations

Increased rod-cone coupling at night not only transmits very dim rod signals to cones and other neurons in cone pathways, but it also improves detection of very dim large objects at night by improving the signal to noise ratio and the reliability of rod responses to very dim light. Biological systems that increase in sensitivity typically become noisier. A considerable challenge for sensory systems is the difficulty detecting faint signals and distinguishing them from noise. Increased photoreceptor coupling at night may thus represent a significant evolutionary innovation. Because intrinsic noise in each photoreceptor cell is independent of the noise in other nearby coupled photoreceptors, but dim objects produce correlated photoreceptor signals, photoreceptor coupling reduces photoreceptor noise more than it reduces their output signals, especially for signals in response to large dim objects (Ribelayga et al., 2008; Jin et al., 2015). The increased coupling between photoreceptors at night therefore enhances the signal to noise ratio and the reliability of rod responses to large dim objects. Moreover, the increase in signal to noise ratio is greater the more cells that are coupled.

It is worth noting that both the extrinsic (D_1_R) and intrinsic (D_4_R) dopamine systems function in a complementary manner and unfold slowly, i.e., _._they only need to keep pace with the rotation of the Earth. In the outer retina, D_1_Rs and D_4_Rs, which have opposite effects on intracellular cAMP/PKA when activated (Kebabian and Calne, 1979; Missale et al., 1998), are located on different cell types, and due to their different affinities for endogenous extracellular dopamine, essentially function at different times of the day. That is, during the night in the dark when only stars are present, the retina clock does not activate D_4_Rs. As a result, rods and cones are coupled, cBC receptive field centers are large, and retinal neurons have high sensitivity to very dim large objects. As night then turns to dawn, the retinal clock slowly increases extracellular dopamine and endogenous activation of cone D_4_Rs. As a result, rod-cone coupling gradually decreases, slowly transforming rod vision to cone vision.

Then, when the ambient light level elicits sufficient dopamine release to activate D_1_Rs, HCs begin to uncouple and ON-cBC dendrites begin to express GABA_A_Rs. This process slowly increases the strength of ON-cBC surrounds. At the same time, as D_1_R activation continues to increase, HCs, which use GABA to send a receptive field surround signal to ON-cBC dendrites, communicate a progressively smaller and smaller surround to ON-cBCs, The reduction in the size of receptive field centers and surrounds of cone-driven BCs and ganglion cells as the maintained background light level increases (Hammond, 1975; Troy and Shou, 2002; Thoreson and Mangel, 2012) suggests the interaction between center and surround spatially differentiates progressively smaller regions of the visual scene as ambient illumination gradually increases. This D_1_R-mediated modulatory process likely enhances the ability of BCs and ganglion cells to discriminate fine spatial detail and edges when maintained illumination is bright.

Finally, it is important to note that the modulatory effects of dopamine in the outer retina are likely conserved across vertebrate species, including mammals and non-mammals. Specifically, circadian pathway components and effects and the location of D_1_Rs and D_4_Rs in the outer retina have been observed in many vertebrate species that contain both rods and cones (Iuvone et al., 2005; Besharse and McMahon, 2016). For example, D_4_Rs (but not D_1_Rs) are located on rods and cones, and D_1_Rs (but not D_4_Rs) are located on the dendrites of cone-connected BCs and HCs (Witkovsky, 2004). In addition, the retinal circadian clock in goldfish, rabbit, and mouse regulates rod-cone coupling *via* D_4_Rs (Ribelayga et al., 2008; Ribelayga and Mangel, 2010). Also, light and dark adaptation, by modulating activation of dopamine D_1_Rs on the dendrites of cBCs and HCs in goldfish and rabbits regulates GABA_A_R expression and activity on the dendrites of these cells (Witkovsky, 2004; Chaffiol et al., 2017). It is therefore likely that the distinct modulatory actions of dopamine D_1_Rs and D_4_Rs in the outer retina discussed in this review can be generalized across species that contain both rods and cones.

## Author Contributions

MG and SM wrote the manuscript. All authors contributed to the article and approved the submitted version.

## Conflict of Interest

The authors declare that the research was conducted in the absence of any commercial or financial relationships that could be construed as a potential conflict of interest.
